# Foraging as an evidence accumulation process

**DOI:** 10.1371/journal.pcbi.1007060

**Published:** 2019-07-24

**Authors:** Jacob D. Davidson, Ahmed El Hady

**Affiliations:** 1 Department Collective Behavior, Max Planck Institute for Animal Behavior, Konstanz, Germany; 2 Centre for the Advanced Study of Collective Behaviour, University of Konstanz, Konstanz, Germany; 3 Department of Biology, University of Konstanz, Konstanz, Germany; 4 Princeton Neuroscience Institute, Princeton, New Jersey, United States of America; 5 Howard Hughes Medical Institute, Chevy Chase, Maryland, United States of America; Santa Fe Institute, UNITED STATES

## Abstract

The patch-leaving problem is a canonical foraging task, in which a forager must decide to leave a current resource in search for another. Theoretical work has derived optimal strategies for when to leave a patch, and experiments have tested for conditions where animals do or do not follow an optimal strategy. Nevertheless, models of patch-leaving decisions do not consider the imperfect and noisy sampling process through which an animal gathers information, and how this process is constrained by neurobiological mechanisms. In this theoretical study, we formulate an evidence accumulation model of patch-leaving decisions where the animal averages over noisy measurements to estimate the state of the current patch and the overall environment. We solve the model for conditions where foraging decisions are optimal and equivalent to the marginal value theorem, and perform simulations to analyze deviations from optimal when these conditions are not met. By adjusting the drift rate and decision threshold, the model can represent different “strategies”, for example an incremental, decremental, or counting strategy. These strategies yield identical decisions in the limiting case but differ in how patch residence times adapt when the foraging environment is uncertain. To describe sub-optimal decisions, we introduce an energy-dependent marginal utility function that predicts longer than optimal patch residence times when food is plentiful. Our model provides a quantitative connection between ecological models of foraging behavior and evidence accumulation models of decision making. Moreover, it provides a theoretical framework for potential experiments which seek to identify neural circuits underlying patch-leaving decisions.

## Author summary

Foraging is a ubiquitous animal behavior, performed by organisms as different as worms, birds, rats, and humans. Although the behavior has been extensively studied, it is not known how the brain processes information obtained during foraging activity to make subsequent foraging decisions. We form an evidence accumulation model of foraging decisions that describes the process through which an animal gathers information and uses it to make foraging decisions. By building on studies of the neural decision mechanisms within systems neuroscience, this model connects the foraging decision process with ecological models of patch-leaving decisions, such as the marginal value theorem. The model suggests the existence of different foraging strategies, which optimize for different environmental conditions and their potential implementation by neural decision making circuits. The model also shows how state-dependence, such as satiation level, can affect evidence accumulation to lead to sub-optimal foraging decisions. Our model provides a framework for future experimental studies which seek to elucidate how neural decision making mechanisms have been shaped by evolutionary forces in an animal’s surrounding environment.

## Introduction

In systems and cognitive neuroscience, decision-making has been extensively studied using the concept of evidence accumulation [1, 2, 3, 4, 5, 6, 7]. Evidence accumulation has been implicated for example in social decisions [8], sensory decisions [9, 10], economic decisions [11], memory decisions [12], visual search decisions [13], and value decisions [14]. Moreover, neural recordings have given the experimenter the opportunity to investigate a myriad of neuronal mechanisms underlying these decision processes [10, 15, 16, 17, 18, 19, 20, 21, 22, 23, 24]. Although this line of work has revealed a detailed account of the neural mechanisms associated with decision-making, an outstanding question remains as to how these mechanisms have been shaped by selection forces in the animal’s environment [25, 26].

Foraging is one of the most ubiquitous behaviors that animals exhibit, as search for food is essential for survival [27]. From a cognitive perspective, foraging comprises aspects of learning, statistical inference, self-control, and decision-making, thus providing the opportunity to understand how these processes have been shaped by natural selection to optimize returns in the face of environmental and physiological constraints and costs [26]. The patch-leaving problem is a canonical foraging task where an animal must decide when to leave a resource to search for another. Ecological models, such as the well-known marginal value theorem (MVT) [28], describe patch-leaving decision rules that an animal should use to optimize its food intake. Deviations from optimal decisions may be due to internal state-dependence or environmental characteristics [29]. Studies that link cognitive biases to environmental structure highlight the importance of studying the decision-maker in their natural environment, by framing decision making in terms of “ecological rationality” (as opposed to “economic rationality”) [30, 31].

There is an increased interest to study foraging behavior within a neuroscience context and link neural signals to relevant foraging parameters [32, 33, 34, 35, 36, 37]. For example, during a visual foraging task with non-human primates (*Macaca mulatta*), the activity in the dorsal anterior cingulate cortex (dACC) region was found to increase while a patch depletes until a threshold, after which the animal switches patches [32]. Other work has found that neurons in primate posterior cingulate cortex (PCC) signal decision salience during visual foraging, and thus relate to disengagement from the current patch [38]. These studies aim to understand the neural mechanisms behind foraging decisions, and how an animal uses its experience to reach patch-leaving decisions. While the MVT provides a quantitative basis for understanding patch decisions in the context of optimal decision-making, it does not give a mechanistic account of the animal’s internal decision process.

In this work we formulate a mechanistic model of patch-leaving decisions by linking ecological models of the patch-leaving task with models of evidence accumulation that are used in systems neuroscience. We call this model the foraging drift-diffusion model (FDDM). This model builds on previous mechanistic models of patch-leaving decisions [39, 40, 41, 42, 43]. In our model, patch-leaving decisions are described by a drift-diffusion process [44, 45], which represents the noisy process through which an animal accumulates evidence (by finding food), and how this experience is used to decide when to leave the patch. Evidence accumulation and decisions within a patch are coupled to a moving average process that keeps track of the average rate of energy available from the environment. We solve for conditions where the model yields optimal foraging decisions according to the MVT, and perform simulations to analyze deviations from optimal when these conditions are not met. We show that optimal decisions can be represented in the model using different decision “strategies”, including an incremental mechanism, where receiving food reward makes the forager more likely to stay in the patch, and a decremental mechanism, where receiving food reward makes the forager more likely to leave. These strategies are adaptive to different environmental conditions, depending on the uncertain versus known information about the foraging environment. To account for the salient experimental observation that patch residence times tend to be longer than optimal, we introduce a marginal utility function into the model and show how this leads to sub-optimal foraging decisions. Importantly, our model generates testable predictions about the different decision strategies an animal may employ in an uncertain environment. The model provides a quantitative connection between foraging behavior and experiments that seek to understand the neural basis of patch-leaving decisions.

## Results

### Foraging drift-diffusion model (FDDM)

The model that we term foraging drift-diffusion model (FDDM) includes two coupled equations. The first is an averaging process to estimate the available energy in the environment [46, 47]. The forager receives rewards according to a time-dependent reward function *r*(*t*), which is zero when outside of a patch. There is a constant cost of *s*, so that the net rate of energy gain while in a patch is *r*(*t*) − *s*, and while traveling between patches it is −*s*. With this information, the energy intake available from the environment (*E*) is estimated by taking a moving average over a timescale *τ*_*E*_:

**Estimate of available energy**
  $$\begin{array}{c} \tau_{E}dE=(r(t)-s-E)dt \end{array}$$ (1)

The second equation provides a mechanistic description of when to leave an individual patch based on the actual experience of rewards [39, 40, 41, 42, 43]. Motivated by models of decision-making [44, 45], we represent this using a drift-diffusion process via a patch decision variable *x*. Upon entering a patch *x* = 0, and changes in *x* occur with evidence accumulation from a constant drift *α* and time-dependent rewards *r*(*t*). The forager decides to leave the patch when the threshold of *x* = *η* is reached.

**Decision to leave a patch**
  $$\begin{array}{c} \tau dx=(\alpha -r(t))dt+\sigma dW\left(t\right), \end{array}$$ (2)

**Fig 1:**
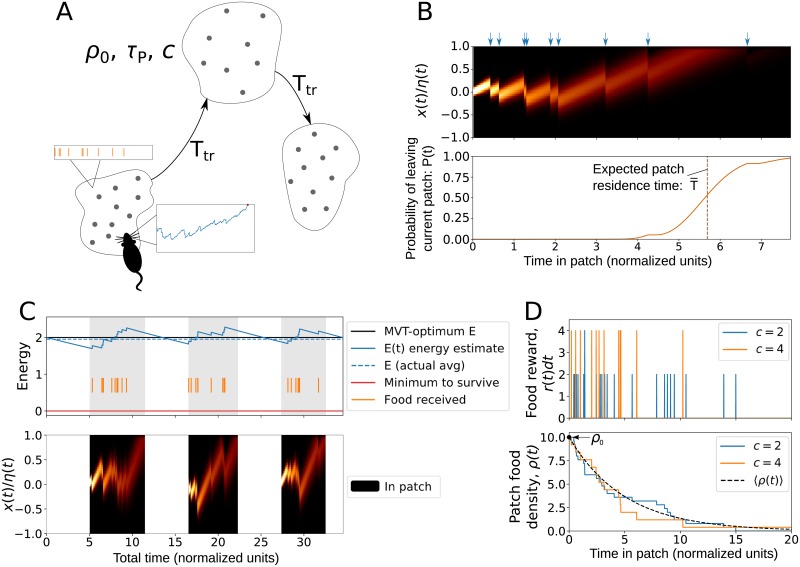
Foraging-drift-diffusion model. (A) Schematic showing the patch-leaving task: A forager estimates the average rate of reward from the environment, and the decision to leave a patch occurs when the internal decision variable reaches a threshold. Travel time between patches is *T*_*tr*_, and patches are described by the parameters *ρ*_0_, *A*, and *c* (see Table 2). (B) Evolution of the probability density of the patch decision variable (*x*) while in a single patch, along with the time-dependent probability that the decision to leave the patch has been made. Blue arrows denote the receipt of food rewards. (C) Energy estimate coupled with the patch decision variable over multiple patches. (D) Patch depletion with discrete rewards, showing examples of the food reward received and the time-dependent in-patch food density for different values of the food chunk size (*c*).

In the following sections, we show that Eq 2 can account for robust patch-leaving decisions in the case of noisy sampling of the environment, and can be generalized to exploit knowledge of the foraging environment. Fig 1 shows a schematic of the model, an example for the probability density of *x* when in a patch, and example traces of *E* and *x* across multiple patches. Table 1 lists the quantities defined in the governing equations.

**Table 1:** Variable definitions for the coupled model formulation in Eqs 1 and 2.

Energy and patch decision variables	
*E*	Estimated environment energy rate
*τ*_*E*_	Timescale for updates of E
*r*(*t*)	Current gross rate of energy (food) intake
*s*	Constant cost
*x*	Decision state for when to leave a patch
*τ*	Timescale for updates of *x*
*α*	Drift rate
*η*	Threshold for decision to leave a patch.
*σ*	Noise for patch decisions
*W*(*t*)	Wiener process

**Table 2:** Variables and parameters used to describe patch quality and depletion.

Patch variables and parameters	
*ρ*(*t*)	Time-dependent food density in the current patch
*ρ*_0_	Initial food density
*A*	Patch size
*c*	Food chunk size
*T*_*tr*_	Travel time between patches

### Food reward and patch characteristics

The function *r*(*t*) describes the rate of food reward that the animal receives while in a patch, and *ρ*(*t*) is the density of food in the current patch. The initial density of food in the patch is *ρ*_0_, and when a forager finds and eats a piece of food, the total amount of food remaining in the patch decreases. To formalize this, we consider that patches have an area of *a* and that food is uniformly scattered within a patch in chunk sizes of *c*. The parameter *c* interpolates between continuous (*c* = 0) and discrete (*c* > 0) food rewards. If the forager searches at a rate of *v*, the probability of finding *k* chunks of food in a time interval Δ*t* is given by a Poisson distribution with event rate of *ρ*(*t*)*v*Δ*t*/*c*. Without loss of generality, we set *v* = 1, i.e. the forager explores one unit area per unit time, and define *A* = *a*/*v* as the “patch size”, with units of time. The average change in patch food density follows a simple exponential decay (see Methods):

$$\begin{array}{c} \left\langle \rho \left(t\right)\right\rangle =\rho_{0}e^{-t/A}, \end{array}$$ (3)

where *t* is the time spent in the current patch. The reward rate is defined as the negative change in patch density,

$$\begin{array}{c} r\left(t\right)=-A\frac{d\rho (t)}{dt}, \end{array}$$ (4)

which can be calculated in the discrete case by considering the change over a discrete time interval Δ*t*. The average reward rate is thus given by the same exponential decay as the average change in patch food density:

$$\begin{array}{c} \left\langle r(t\right)\rangle =\rho_{0}e^{-t/A}. \end{array}$$ (5)

Fig 1D shows example time traces of patch density and food received for different values of the chunk size *c*. In limit of zero chunk size, food reward is continuous and the food reward rate and patch density are equal to the average density:

$$\begin{array}{c} \lim_{c\to 0}r\left(t\right)=\lim_{c\to 0}\rho \left(t\right)=\left\langle \rho \left(t\right)\right\rangle . \end{array}$$ (6)

### Optimal foraging and patch decision strategies

We solve the model to establish conditions on the drift rate *α* and the decision threshold *η* which lead to approximately optimal patch residence times. To obtain these conditions, we analytically treat the simplified case of *E* = 〈*E*〉 = *const*., (the estimated value of energy is constant and equal to the actual average), *σ* = 0, (no noise on the patch decision variable), and *c* = 0 (food reward is received continuously). We then relax these assumptions using simulations with an evolving, time dependent estimate of available energy (*E*), and show that the derived rules lead to approximately optimal patch decisions over a wide range of parameter values and configurations of the foraging environment.

First, we rewrite the marginal value calculation for patch residence time (PRT) using the above notation. If there is a travel time between patches of *T*_*tr*_, then the average rate of energy intake is given by a weighted sum of intakes during time in and traveling between patches. Taking the derivative of the average energy intake rate, setting to zero, and re-arranging, yields the well-known condition to solve for the MVT-optimal time T* to stay in a patch:

$$\begin{array}{c} \underset{\text{marginal}\;\text{in-patch}\;\text{rate}}{\underset{︸}{r(T^{*})-s}}=\underset{\langle E\rangle =\text{average}\;\text{energy}\;\text{rate}}{\underset{︸}{\frac{\int_{0}^{T^{*}}r\left(t\right)dt-s*\left(T_{tr}+T^{*}\right)}{T_{tr}+T^{*}}}} \end{array}$$ (7)

Eq 7 can be written compactly as *r*(*T*) − *s* = ⟨*E*⟩, where ⟨*E*⟩ is the average energy rate from the environment. If rewards are continuous, e.g. following Eq 6, then the condition *r*(*T*) − *s* = ⟨*E*⟩ can be used instead of Eq 2 as a decision rule for when to leave a patch; indeed, this is the original rule defined by the marginal value theorem [28]. However, when rewards are stochastic, the sampling process defined by Eq 2 is needed in order to accurately assess the current level of rewards remaining in the patch.

The optimal time to remain in a patch, according to the MVT, is obtained by inserting the average reward rate (Eq 5) in Eq 7:

$$\begin{array}{c} T^{*}=A\ln\frac{\rho_{0}}{\langle E\rangle +s}. \end{array}$$ (8)

Since the MVT assumes continuous rewards, the patch residence time defined by Eq 8 is not necessarily optimal when rewards are stochastic or discrete; we therefore refer to T* as the MVT-optimal patch residence time, and note (as shown in subsequent sections) that this is not purely optimal when the assumptions of the MVT do not hold. Integrating the patch decision variable (Eq 2) to the threshold and inserting Eq 8 yields a relationship between the threshold, drift rate, energy, and patch parameters:

$$\begin{array}{c} \eta =A(\alpha \ln(\frac{\rho_{0}}{\langle E\rangle +s})-\rho_{0}+\langle E\rangle +s). \end{array}$$ (9)

If Eq 9 is satisfied, MVT-optimal decisions can be obtained with different values of the drift rate, *α*. To define a valid range for *α* values, we require that there is only a single threshold crossing up to the time T* (see Methods), and also omit the small range where *α* and *η* have opposite signs. With this we highlight the following different “strategies”:

$$\begin{array}{cc} \text{Density-adaptive}:\;\; & \alpha =\rho_{0}\\ \text{Size-adaptive}:\;\; & \alpha =\frac{\rho_{0}-E-s}{\ln\frac{\rho_{0}}{E+s}}\\ \text{Counting}:\;\; & \alpha =0\\ \text{Robust}\;\text{counting}:\;\; & \alpha <0. \end{array}$$ (10)

For each strategy, *η* is defined by Eq 9 with the corresponding value of *α*, and substituting *E* instead of ⟨*E*⟩.

Patch decision strategies can be placed in two categories depending on the signs of *α* and *η*, which give qualitatively different results because evidence accumulation is the difference between drift and reward. When *α* > 0 and *η* ≥ 0, this is an increment-decrement, or incremental, mechanism [41, 48], which in previous work has been suggested as adaptive for the case when the forager does not initially know the number of expected reward items on the patch [49]. Finding food decreases *x*, and makes the forager more likely to stay in the patch, but otherwise drift increases *x* towards the positive threshold value. Thus, drift and food reward have opposite effects on how long the forager stays in a patch.

**Fig 2:**
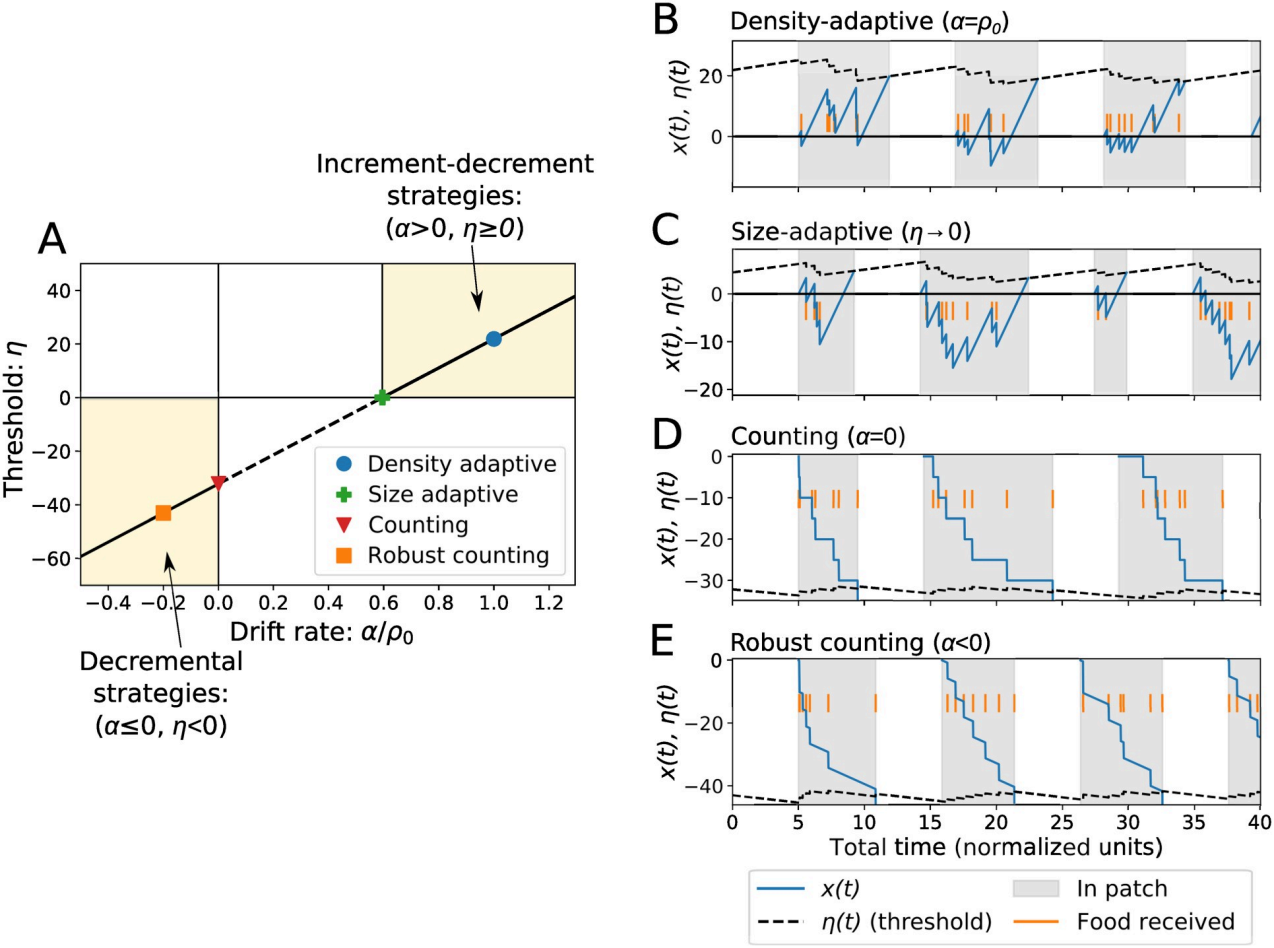
Patch-leaving decision strategies. Different strategies are represented with different choices of the drift rate (*α*) and the threshold (*η*) (Eq 10). (A) The optimal threshold from Eq 9 plotted as a function of the drift, showing the general classes of increment-decrement and decremental strategies. The solid line shows the optimal threshold in the region of parameter space where both *α* and *η* have the same sign, and the dotted line indicates the region where they have the opposite sign. Parameters used are *A* = 5, E* = 2 (or equivalently, *ρ*_0_ = 9.439), and *s* = 2; these parameters are also used in (B-E), which illustrate each strategy using discrete rewards and zero noise on the decision variable. (B) The choice *α* = *ρ*_0_ is optimal for uncertainty in patch food density; this represents an “increment-decrement” mechanism for patch decisions. (C) A threshold of zero is optimal for uncertainty in patch size. Since *η* = 0 is sensitive to noise, we choose a small value *η* > 0 to illustrate. (D) The counting strategy uses zero drift, so that the forager leaves after a set amount of food rewards (E) The robust counting strategy uses *α* < 0 so that there is still drift towards the threshold. Each plot shows the patch decision variable along with the time-dependent patch decision threshold that changes with receipt of food reward due to updates of energy estimate.

The density-adaptive and size-adaptive strategies use an increment-decrement mechanism. The density-adaptive strategy (Fig 2B) sets *α* = *ρ*_0_, which is optimal to adapt PRTs to uncertain food density within each patch (Methods). With this, instantaneous evidence accumulation in Eq 2 is given by (*ρ*_0_ − *r*(*t*)), which can be interpreted as the difference between initial and current reward rate in the patch. Thus, on average, this causes the patch decision variable *x* to always increase towards the decision threshold; first slowly, and then more quickly as the patch becomes depleted. The size-adaptive strategy (Fig 2C) uses a drift value that is optimal to adapt PRTs with respect to uncertainty in the size of each patch (see Methods). The size-adaptive value of *α* sets a threshold of *η* = 0. Since this choice causes *x* to first decrease below zero and then rise back to the threshold, this strategy is sensitive to noise and randomness in the timing of rewards received. We therefore illustrate the size-adaptive strategy in Fig 2C by choosing a value of *α* slightly higher than Eq 10, such that *η* > 0.

The second category of decision strategies is when *α* ≤ 0 and *η* < 0; this represents a ‘decremental’ mechanism of patch decisions [41], where finding food makes the forager more likely to leave the patch. Here, both finding food and drift have the same effect: they decrease *x* towards the negative value of the threshold. The counting and robust counting strategies use a decremental mechanism. The counting strategy has zero drift rate, such that the forager leaves only after a set amount of food reward has been received (Fig 2D). Since the choice of *α* = 0 can lead to infinite PRTs if patches do not contain as much food as expected, we define the additional strategy termed ‘robust counting’ which has a nonzero drift *α* < 0. With a negative value of *α*, there is still drift towards the threshold in the absence of food reward (Fig 2E).

The size-adaptive and counting strategies represent limiting cases of *η* = 0 and *α* = 0, respectively, and this makes these choices sensitive to noise. We therefore focus our analysis on the density-adaptive and robust counting strategies, which both have drift values towards the threshold but differ in how food affects the probability of staying in the patch. Patch decisions using these strategies are exactly equivalent to the marginal theorem for the case of *E* = 〈*E*〉, *σ* = 0, and *c* = 0. In the next section we use simulations to compare model results to MVT optimal behavior for a range of parameter values when *E* ≠ 〈*E*〉, *σ* > 0, and *c* > 0.

### Parameter dependence: Noisy evidence accumulation and discrete food rewards

In the general case, accumulation of evidence will be noisy, food may come in discrete chunks, the estimate of available energy in the environment will vary as the forager explores and obtains food rewards, and patches may vary in quality and distribution. We investigate both a range of environmental configurations and patch parameters as well as different patch decision strategies. To simplify model analysis, we use *τ* as the unit of time, and *s* as the unit of energy, and set *τ*_*E*_ = 50*τ* to represent that the energy estimate occurs at a longer time scale than individual patch decisions. We illustrate dominant trends by choosing an intermediate range for characteristics of the foraging environment: E* = 2*s*, *A* = 5*τ*, and *T*_*tr*_ = 5*τ*. Note that the average energy level is set by using Eqs 7 and 8 to solve for the value of *ρ*_0_ that leads to a certain energy level, given the values of the other parameters, and then using this value of *ρ*_0_ in the simulations.

**Fig 3:**
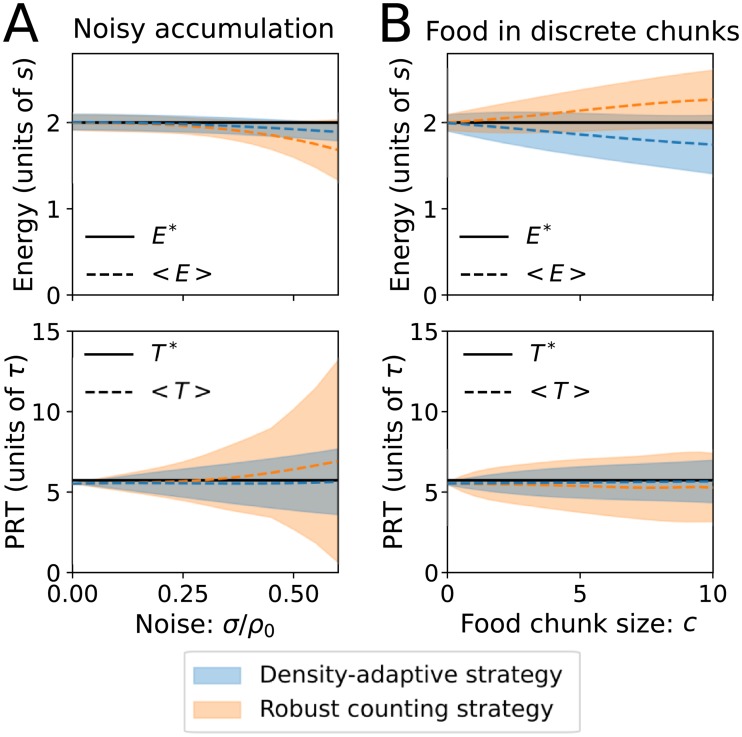
Noisy evidence accumulation and discrete food rewards. Shown are the average and standard deviation of the energy intake and patch residence times, simulated using intermediate values of the patch parameters: *A* = 5, *T*_*tr*_ = 5, and E* = 2 (or equivalently, *ρ*_0_ = 9.439). The filled blue curves use the density-adaptive strategy, and the filled orange curves use the robust counting strategy. The robust counting strategy simulations use *α* = −0.2*ρ*_0_. (A) Simulation results when the noise on the patch decision variable (*σ*) is increased. (B) Simulation results when the food chunk size (*c*) is increased. Analogous simulation results for a range of different parameter values (see Methods) are shown in S1 and S2 Figs.

Fig 3A shows that for small increases of noise on the patch decision variable, both the mean energy intake and mean patch residence time stay near MVT-optimal values, but the variance of patch residence time increases. With zero noise, the mean simulated PRTs are slightly lower than optimal due to the finite time scale for the moving average estimate of energy; *E* tends to be slightly higher than the actual average energy when the agent leaves the patch (e.g. see Fig 1C), which causes the threshold to decrease in magnitude before the forager leaves the patch (see Fig 2). With higher noise values, the average energy intake decreases, and the effect is larger for the robust-counting (RC) strategy compared to the density-adaptive (DA) strategy. With the DA strategy, the variance of patch residence time increases with noise, but the average stays nearly the same. With the RC strategy, the variance increases more strongly with noise, and for large values of *σ*, average patch residence times are longer than optimal.

Fig 3B shows average energy intake and patch residence time when the food chunk size (*c*) increases. For both strategies, larger chunk sizes increase the variance of PRTs without much effect on the mean. However, the two strategies show opposite trends for average energy intake: with the DA strategy, average energy decreases for large chunk size, but with the RC strategy, average energy *increases* for large *c*, to values that are higher than the optimum determined by the marginal value theorem. This is why we refer to E* and T* as “MVT-optimal”, instead of just “optimal”. Using a counting strategy in the case of discrete rewards can lead to energy intakes higher than MVT-optimal because patch-leaving decisions tend to occur immediately after receipt of a food reward, instead of after a certain amount of time in the patch (Fig 2).

With large chunk sizes, the number of food chunks per patch will be small, and therefore instantaneous food intake and leaving decisions are not well described by a ‘rate’, as expressed with the MVT. The optimum number of food chunks obtained per patch is

$$\begin{array}{c} N_{opt}=\frac{A}{c}(\rho_{0}-E-s). \end{array}$$ (11)

For example, using parameter values from Fig 3, a chunk size of *c* = 8 leads to *N*_*opt*_ = 4.02. In this case it is difficult to assess current food density, which is why average energy intake with the DA strategy is less than MVT-optimal (Fig 3B). For extreme cases where *N*_*opt*_ < 1, which occurs for example with small patch size, short inter-patch travel times, and low available energy in the environment, the DA strategy performs poorly, while the RC strategy yields average energy intake rates that are higher than MVT-optimal (S2 Fig).

### Patch uncertainty and adaptive decisions

To this point we have considered cases where patch quality and inter-patch travel times are the same for all patches; we now ask how the different strategies perform when aspects of the foraging environment are uncertain and may vary from patch to patch. The MVT predicts that foragers should stay longer in high quality patches, and shorter in low quality patches. However, this assumes that as they enter a patch, the forager recognizes the ‘type’ of the patch and therefore adjusts their expectation of food rewards. We instead consider that the forager only knows the *average* patch quality in the environment, and must use this along with the estimate of *E* and its current experience of food rewards to determine when to leave a patch.

**Fig 4:**
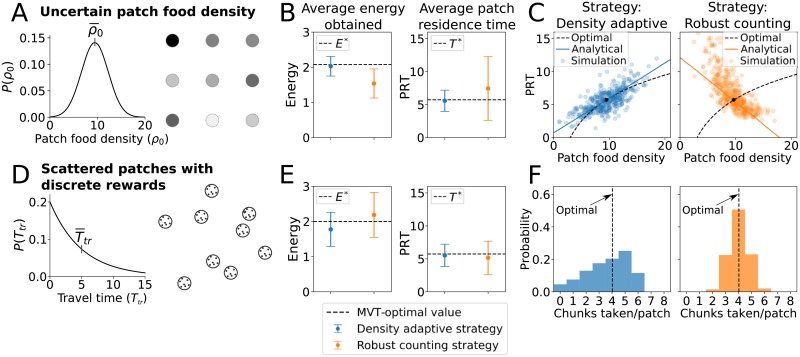
Different foraging environments with associated patch decision strategies. Shown are simulation results with the density-adaptive and robust-counting strategies in two different foraging environments. (A,D) illustrates the foraging environment for a given case, (B,E) shows average energy and patch residence time when a particular strategy is used in that environment along with the MVT-optimal energy energy (E*) and patch residence time (T*), and (C,F) shows simulation results compared to MVT-optimal strategies in each environment. All simulations use a noise level of $\sigma =0.3\bar{\rho_{0}}$ and a patch size of *A* = 5, and the robust counting strategy is implemented by setting *α* = −0.2*ρ*_0_. (A-C) Uncertainty in patch food density. Patches have a Gaussian distribution for initial food density with mean of $\bar{\rho_{0}}=9.439$ and a standard deviation of $\Delta \rho_{0}=0.3\bar{\rho_{0}}$, and rewards are received continuously (*c* = 0). Travel time between patches is constant at *T*_*tr*_ = 5. The solid line in (C) shows an approximate analytical solution (Methods, Eq 25) for small changes in *ρ*_0_ about $\bar{\rho_{0}}$. (D-F) Scattered patches with discrete rewards. Food reward is received in discrete chunks (*c* = 8) and each patch has the same initial food density of *ρ*_0_ = 9.439. Travel time between patches is drawn from an exponential distribution with mean $\bar{T_{tr}}=5$. (F) shows a histogram of simulation results for how many food chunks were taken before leaving the patch, for both the density adaptive strategy (left) and the robust counting strategy (right).

We first consider a case where patch quality is uncertain, by varying the initial food density of each patch. Using the DA strategy in the model, foraging decisions follow the same trend as the MVT: foragers stay longer in higher quality patches (i.e. patches with higher *ρ*_0_) and shorter in lower quality patches (i.e. lower *ρ*_0_). In contrast, the RC strategy yields the opposite trend: patch residence time *decreases* with patch quality (Fig 4C). Therefore, in this environment, while the DA strategy yields an average energy intake and PRT close to optimal, using the RC strategy yields an energy intake lower than optimal (Fig 4B) due to the increase in PRTs.

We next consider a different configuration of the foraging environment: food is received in discrete chunks, patches are randomly distributed about the landscape, but the quality of each patch is the same. Because each patch contains the same amount of food, an optimal strategy is to ‘count’, i.e. to leave a patch after a certain amount of food reward is received. Simulations with noise show that in this environment, the RC strategy leads to a higher average energy intake than the DA strategy (Fig 4E). This is because the distribution of number of food items per patch is sharply peaked near the optimal value for the RC strategy, while the distribution is broader with the peak skewed from optimal for the DA strategy (Fig 4F). Similar to Fig 3B, Fig 4E shows that the RC strategy leads to mean energy intakes that are higher than the optimum predicted by the MVT, because patch-leaving decisions tend to occur immediately following the receipt of food reward.

Another type of patch uncertainty can come from patches that vary in size. The size-adaptive (SA) strategy defined in Eq 10 yields adjustments to PRTs based on the size of each patch that follow, in the limiting case of zero noise, the optimal times given by Eq 8. However, because the SA strategy has a threshold of zero, it is very sensitive to noise. In simulations with added noise, using a small but nonzero threshold (i.e. values close to the SA strategy) yields similar or slightly lower average energy intakes compared to the DA strategy when patch size is uncertain (S3 Fig). This suggests that while a forager with an appropriate strategy can nearly optimally adapt individual patch residence times to uncertainty in patch food density, it is more difficult to use a noisy sampling process to adapt individual patch residence times to uncertainty in patch size.

### Sub-optimal behavior: Satisficing

With the exception of the RC strategy in an environment where patch quality is uncertain, simulations yield average PRTs that are near or slightly lower than optimal. Many studies have examined patch residence times in comparison to MVT predictions; the most common trend is that animals tend to stay *longer* in patches than predicted by the MVT [29]. In this section we introduce a change to the model to account for this observation.

An animal’s perception of a reward, and subsequent foraging decisions, depend on their internal state. One way to capture this is by using a utility function approach, borrowed from behavioral economics [50, 51]. This is also related to ‘satisficing’ [52, 53, 54], defined as the process by which animals do not seek to maximize food intake, but instead seek to maintain food intake above a threshold. If food is plentiful, then the marginal utility of increasing intake is small; in this case, an animal will likely be more concerned with, for example, avoiding threats than leaving a current patch in search of higher returns. Conversely, if food is scarce, then survival depends on maximizing the rate of food rewards.

We model this by introducing a function *u*(*E*) for the marginal utility of additional rewards, which depends on *E*, which is the forager’s time-averaged energy intake from the environment. The utility function modifies patch decision dynamics by changing the drift rate and the impact of receiving food:

$$\begin{array}{c} \tau dx=(\alpha u\left(E\right)-r\left(t\right)u{\left(E\right)}^{-\text{sgn}(\eta )})dt+\sigma dW\left(t\right). \end{array}$$ (12)

Using this form, the utility function decreases the rate of drift towards the threshold, and either increases or decreases the change in *x* with food reward depending on whether the threshold is positive or negative. To define *u*(*E*), first recall that the animal must obtain energy *E* > 0 in order to survive. In the limit *E* → 0, we therefore expect that an animal will adopt a foraging strategy that maximizes energy intake; this is set by *u*(0) = 1. For high values of *E*, we expect that the animal cares less about maximizing food intake rate, and therefore *u* should decrease. We consider two functions to represent this:

$$\begin{array}{c} u_{\text{exp}}\left(E\right)=\left(1-A\right)e^{-\beta E}+A \end{array}$$ (13)

$$\begin{array}{c} u_{\text{lin}}\left(E\right)=\{\begin{array}{cc} 1-\beta E & \text{if}\;1-\beta E\geq A\\ A & \text{if}\;1-\beta E<A \end{array}, \end{array}$$ (14)

where *β* > 0 is a parameter that determines how fast the marginal utility changes with energy. An approximate solution for how the marginal utility function affects energy intake and PRT is obtained by integrating Eq 12 using either Eqs 13 or 14, setting *σ* = 0 and *E* = ⟨*E*⟩, and combining with Eqs 7 and 8. Note that Eqs 13 and 14 are marginal utility functions, i.e. the change of utility with respect to changes in energy, and the full utility function can be obtained by integrating with respect to *E*. We chose the exponential and threshold linear forms for *u*(*E*) to investigate the model response, and note that other functional forms can be used.

**Fig 5:**
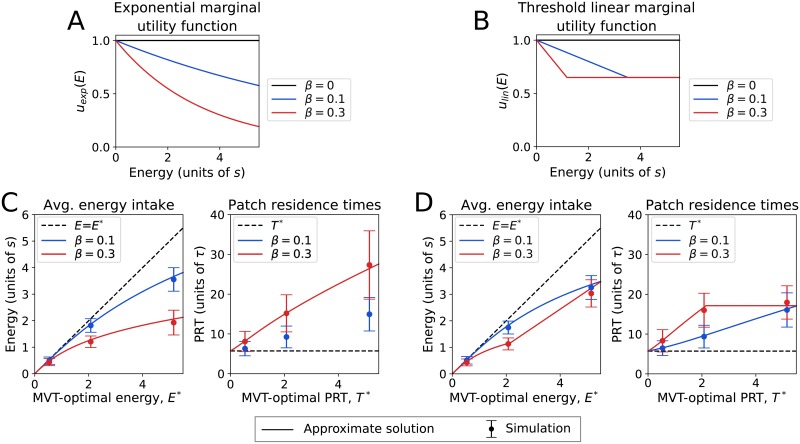
Sub-optimal behavior. The marginal utility of additional food reward may depend on the current rate of energy intake. We consider two possible functions: (A) Exponential decreasing utility, shown using *A* = 0 in Eq 13. (B) Threshold linear decreasing utility (Eq 14), shown here using a threshold of 0.65. Each form of the utility function has a parameter *β* that sets how fast the utility decreases with energy. Simulation results using the exponential utility function are shown in (C), and corresponding results using the threshold linear utility function in (D). For each case of the utility function, the average energy intake and patch residence time are shown for two different values of *β*. Solid lines are an approximate solution to the governing equations and points are the mean and standard deviation of simulation results. Both (C) and (D) use the density-adaptive strategy, and an environmental configuration where patch food density is uncertain (i.e. the same configuration and parameters as Fig 4A–4C). Analogous results for the robust counting strategy, and an additional environmental configuration, are shown in S4 Fig.

Using either form of the marginal utility function leads to patch decisions that approach optimal when energy is low, but deviate from optimality when energy is high, in particular for the larger values of *β* (Fig 5). Although both forms of the utility function demonstrate longer than optimal patch residence times, the change of PRTs with energy levels depends on whether the exponential or threshold linear form is used.

## Discussion

In this study, we developed a foraging drift-diffusion model (FDDM) to describe how an animal accumulates evidence over time in the form of food rewards and uses this experience to decide when to leave a foraging patch. Our model links ecological models of patch foraging with drift-diffusion models of decision making. We solved for conditions where the FDDM yields identical decisions to the marginal value theorem, and performed simulations to show how deviations from optimality are affected by noisy evidence accumulation and discrete versus continuous food rewards. By adjusting the drift rate and the threshold for patch decisions, the model can represent different decision strategies, including an increment-decrement (or incremental) mechanism, where finding food makes the animal more likely to stay in the patch, and a decremental mechanism, where finding food makes the animal more likely to leave the patch. We obtained approximate solutions in addition to model simulations to demonstrate how these different strategies are adaptive, depending on the known and unknown aspects of the foraging environment. We then showed that incorporating a utility function into the model can quantitatively account for the common experimental observation that patch residence times tend to be longer than optimal.

The FDDM model builds on a body of previous work that has considered statistics of patch depletion [55, 56], averaging mechanisms to estimate available energy [46, 47], and “patch-leaving potentials” or other mechanistic descriptions of when to leave a patch [39, 40, 41, 42, 43]. The FDDM model combines these different mechanisms into a single model with a tractable analytical form, and establishes a framework for future experiments that seek to understand different decision strategies that may depend on environmental characteristics, neural dynamics, and state-dependence of the animal. In previous studies, the “recent experience-driven model” considered a finite timescale for updates of energy [47], and another approach represented the forager as estimating the average “profitability” of the environment [46]. Mechanistic models of patch-leaving decisions have proposed that a forager has a “patch potential”, which declines in the absence of food and increases when food is found, and then the forager leaves when the potential crosses zero [39, 40]. Other work has modeled patch-leaving decisions by considering a leaving potential [41, 42], or the probability of continuing to stay in the patch for a certain amount of time [43]. We note that all of these models, as well as the FDDM, can represent similar patch decision mechanisms: the increment-decrement, or incremental, mechanism refers to when finding food makes the forager more likely to stay, and the decremental mechanism refers to when finding food makes the forager more likely to leave [41]. We showed that the counting strategy is a special case of the decremental mechanism for patch-leaving decisions. We note that although we used a drift-diffusion process to describe patch-leaving decisions, an alternative formulation could use an Ornstein-Uhlenbeck process to represent leaky accumulation of evidence [57]; these two models will yield similar results when the time scale for leak is small compared to the patch residence times.

The utility-function approach represents foraging decisions which lead to sub-optimal energy intake and longer than optimal patch residence times. This formulation relates to the mechanisms of temporal discounting and satisficing. Temporal discounting, (also called ‘delay discounting’), refers to when the animal values current rewards more than expected future rewards [58, 59, 60]. Satisficing refers to when animals do not seek to maximize food intake, but instead to maintain food intake above a threshold [52, 53, 54]. Various models of cognitive biases have incorporated these mechanisms to explain cases where the forager stays in a patch longer than optimal. One way to model a bias is by discounting of future rewards, so that for example an expected large reward in a new patch is discounted because of the time delay until which it is available [61]. An alternative model uses a decreasing marginal utility function, such that an expected large reward in a new patch is not viewed as proportionally better than the current low rate of reward in an almost-depleted patch, for example due to costs associated with switching patches [62]. Another possibility is to define a subjective cost that approximates the aversion to leave the patch [63, 64]. In our model, the utility function can be interpreted as a satisficing mechanism; if food is plentiful, then the marginal utility of leaving the current patch to search for a new patch with possibly higher rewards is small, and therefore the animal stays longer in the current patch. The reason for this could be that the animal is satisfied with its current rate of food intake, or that due to other factors (e.g. risks involved with continued search), it values receiving smaller, certain rewards in the present moment instead of leaving to obtain uncertain but possibly larger rewards. We investigated two examples for the form of the marginal utility function in Fig 5, and note that an interesting area for future work is to ask how an animal’s perception of the value or utility of a reward depends on internal state and external environmental conditions. We also note that because both the density adaptive and robust counting strategies display longer than optimal patch residence times when the utility function is introduced (S4 Fig), it would be difficult to use only patch residences times to distinguish what strategy an agent is using.

Foraging decisions differ from common models of economic choice in a key aspect: decisions are sequential, instead of between discrete alternatives [65]. Experiments with the “self-control preparation”, where an animal must choose between two alternatives, and the patch preparation, which is a sequential foraging preparation, have seen behavioral differences even though from an economic standpoint the setups are equivalent [59]. Considering stay-or-go choices instead of choices between alternatives is also different from the modeling perspective. A fundamental aspect of the two-choice decision task is the speed-accuracy tradeoff: this describes that the longer one waits to make a decision (and thus is able to accumulate more evidence), the more accurate the decision will be. It also means that easy decisions tend to be made more quickly than difficult decisions. The two-boundary drift-diffusion model is an optimal strategy to balance the speed-accuracy tradeoff, in that it acheives the fastest decisions for a given level of accuracy (or vice versa) [6, 45]. However, the speed-accuracy tradeoff does not apply to a stay-or-go task such as patch-leaving, where the decision itself is how long to stay in the patch. In psychology, stay-or-go decisions have been investigated with a “go/no-go” task, where subjects must respond to one alternative while withholding a response to the other. Similar to the patch-leaving task, the go/no-go task requires the subject to decide on a response time. However, since a second alternative still exists (but choices to it are suppressed), this makes it different from foraging. Casting patch-leaving decisions into a similar context would require giving the forager knowledge of the two future patches A and B, but then not allowing choices to patch B. Because of this task difference, go/no-go experiments are better fit with an “implicit lower boundary” model instead of a model with a single boundary [66]. Since we assumed that the forager must search to find the next patch, and subsequent patches are “reset” each time, independent of the previous patch, the decision to leave was modeled based only on experience at the current patch. An extension to the FDDM could consider a forager which keeps memory of a multi-patch environment. In this case, decisions could be based both on the experience at the current patch, and on specific knowledge of the reward contents of other patches that are nearby.

In this study we modeled a single forager acting independently. Often times a more realistic situation involves other agents who simultaneously exist in the environment, which leads to competitive and/or collective foraging. If foragers are competing for resources, the ideal free distribution theory describes an optimal way to distribute multiple agents at different food sources in relation to the quality of food sources and the density of competition [67]. Other work has asked how competition between foragers may drive differences in individual strategies, and how different foraging strategies are related to heritable genetic variation in *C. Elegans* [68]. C. Elegans foraging has ‘burst’ and ‘pause’ periods, and a drift-diffusion modeling approach has previously been used to analyze how decisions adapt to changing environmental conditions [69]. Considering a competitive environment, the FDDM could be used to simulate multiple agents who may occupy patches at the same time. In such a case, an individual’s personal experience of food rewards may not accurately reflect actual patch quality, because of the simultanous depletion of the patch between agents who may not communicate internal state or receipt of rewards. It would be interesting to compare this to the cases of uncertain patch quality that we simulated with an individual forager.

In other cases a group may forage together collectively, leading to individual decisions that incorporate both non-social and social information (e.g. [70]). Patch-leaving decisions will then depend on the group reaching consensus. The drift-diffusion modeling framework has been extended to represent coupled decision-makers who share information to collectively reach a decision [71], and this approach could be used to extend the FDDM to multiple agents who make decisions as a group.

We considered that the forager knows the average patch food density ($\bar{\rho_{0}}$) and the average patch size ($\bar{A}$), and uses these to set an optimal decision “strategy” by choosing values of the drift rate (*α*) and threshold (*η*). Other models have considered the process of learning about the environment during foraging using reinforcement learning [72]. Reinforcement learning (RL) is a framework to represent how an agent that receives information about the state of the world along with a scalar valued reward signal learns to select actions which maximize the long run accrued reward. Kolling and Adam [72] reframed the MVT rule as an average reward RL algorithm, which estimates relative values of staying and leaving using a particular assumption about the patch’s reward rate dynamics. To incorporate RL into the FDDM, one possibility is that the agent has to learn the patch characteristics $\left(\bar{\rho_{0}},\bar{A}\right)$, and then uses these learned values to set *α* and *η*. Another possibility is that the agent could adjust *α* and *η* directly, based on feedback from the amount of reward received.

Bayesian foraging theories have considered how patch foraging decisions should be based on a prior estimate of the distribution of patches and expected reward in the environment [73, 74]. For example, if it is known that patches contain a set number of reward items, then finding a prey item should decrease the probability of staying at the patch. Conversely, if patch quality is unknown or variable, finding a food item should increase the probability of staying in the patch. These different decision mechanisms correspond to the robust-counting and density-adaptive strategies, respectively. Experimental work has shown that bumblebees make exactly this adjustment to their patch-leaving strategies [75], but bluegill fish do not [76]. Other studies have considered the effect of reward uncertainty (e.g. [77, 78]), suggesting that foragers may not follow optimal rules when patch quality is uncertain [79]. From our simulation results, one possible explanation for sub-optimal decisions when the foraging environment is uncertain is adopting the “wrong strategy” (Fig 4).

In the FDDM, the forager has memory of its previous foraging experience through the estimate of available energy. We note that if the available energy in the environment is known, and does not need to be estimated, then Eq 1 can be omitted by setting *E* = ⟨*E*⟩; we took this approach to analytically solve for approximately optimal decision strategies. Although we did not consider it here, the general coupled form of the FDDM in Eqs 1 and 2 can be used to ask how foraging decisions adapt when the environment changes over time. Previous work has shown that changing environmental conditions can lead to biases from contrast effects [78], the speed of environmental fluctuations affects which strategy is optimal [80], and the relative importance of taking different adaptive strategies depends on the dynamics and predictability of the environment [56]. Spatio-temporal autocorrelation is a common feature of natural environments, and this may have driven certain observed decision biases [81]. Related to this, work has shown that patch time allocation is influenced by recent experiences of travel time [82, 83, 84], and patch quality [85, 86, 87].

In summary, in this work we developed a mechanistic model of a natural behavior (foraging), with a mathematical form inspired by models used in systems neuroscience. This work provides a step towards establishing a unifying framework tying concepts from systems neuroscience, ecology and behavioral economics to study naturalistic decision making. With the advent of functional imaging [88] and wireless electrophysiological techniques in freely moving animals [89], one can monitor different brain areas simultaneously along with the detailed movement and postural dynamics of the animal [90], with the aim to map the involvement of both neurobiological and biomechanical mechanisms that relate to certain aspects of behavior. Additionally, recent advancements in closed loop techniques allow precise perturbations of neural systems that depend on the state and current behavior of the animal [91]. The proposed model provides a moment-by-moment estimate of the evolution of the decision process, which enables future work to map brain activity to quantitative behavioral variables using neural recordings and targeted perturbations.

## Methods

### Simulation details

Patch-leaving decisions with noisy accumulation of evidence (Eq 2) can be simulated by either solving the first passage problem for the probability density, or by generating patch trajectories by simulating a stochastic process. To create Fig 1 we numerically solved the Fokker Planck equation for the probability density of the patch decision variable using the finite element method (Supplemental Section S1). In Fig 1C, patch decisions were coupled to the energy estimate by using the expectation value of the patch residence time; note that alternatively, individual patch decisions could be coupled with the energy estimate by sampling from the solution for the probability distribution of patch residence times.

To obtain the results shown in other figures, we simulated individual decision trajectories by generating random additive noise and a timestep of *dt* = 0.01*τ*. For all results where an average is shown, each case was simulated for a total time of 20000*τ*. To ensure results did not depend on initial conditions, averages were computed by starting from *t* = 1000*τ*. Additionally, we ensured that the averaging during the time 1000*τ* < *t* < 20000*τ* included a ‘full cycle’, by starting the average in a patch and ending after travel between patches.

All simulations were coded in Python.

### Patch depletion

The probability of finding *k* chunks of food of size *c* in a patch with food density *ρ*(*t*) during a time interval Δ*t* by a forager searching at a rate *v* is given by the Poisson distribution:

$$\begin{array}{c} P_{k}=\text{Poisson}(\frac{\rho (t)v\Delta t}{c},k). \end{array}$$ (15)

When food is found, the total amount of food remaining (*aρ*) is reduced by an amount *kc*. On average, the total amount of food, *aρ*(*t*), changes according to

$$\begin{array}{c} a\langle \rho (t+1)\rangle \to \;a\langle \rho (t)\rangle -\langle E\rangle c, \end{array}$$ (16)

Using Eq 15, the average number of pieces of food found in one time step is ⟨*E*⟩ = *ρ*(*t*)*v*Δ*t*/*c*, where 〈⋅〉 denotes an ensemble average. With this, average change in density follows a linear differential equation [55]:

$$\begin{array}{c} A\frac{d\langle \rho \rangle }{dt}=-\left\langle \rho \right\rangle , \end{array}$$ (17)

where *A* = *a*/*v* is the effective time constant of the patch as defined in the text. Without loss of generality, we set *v* = 1, i.e. the forager explores one unit area per unit time. The solution of Eq 17 is the exponential decay given in Eq 3.

### Parameter values for different environmental configurations

In the main text we focused on the intermediate parameter values *A* = 5*τ*, *T*_*tr*_ = 5*τ*, and *E* = 2*s*. To investigate the full parameter dependence of the model, we consider scenarios that represent different configurations of the environment:

1. **Low, medium, and high available energy rates**. The animal needs to obtain energy *E* > 0 to survive. We therefore consider three regimes of the amount of energy surplus available from the environment, defined by considering the MVT-optimal energy in the environment: low (E* = 0.5*s*), medium (E* = 2*s*), and high (E* = 5*s*).
2. **Short, medium, and long inter-patch travel times**. We consider this by using three values for travel times: short (*T*_*tr*_ = *τ*), medium (*T*_*tr*_ = 5*τ*), and long (*T*_*tr*_ = 10*τ*)
3. **Small vs large patches**. A small patch will be depleted quickly, and a large patch will be depleted slowly. We consider small patches with *A* = 1.5*τ*, and larger patches with *A* = 5*τ*.

In all simulations, we set the energy level by using Eqs 7 and 8 to solve for the value of *ρ*_0_ that leads to a certain MVT-optimal energy level, given the values of the other parameters. Simulation results analogous to Fig 3 for the full range of environmental parameters listed here are shown in S1 and S2 Figs.

### Range for drift rate values

Here we determine the values of the drift rate *α* that lead to valid model behavior, defined by where there is only a single threshold crossing during the time 0 < *t* < T*. Let *α*_*S*_ be the drift value of the size-adaptive strategy as defined in Eq 10. Using *α*_*S*_ yields a threshold of *η* = 0. For this case, the patch decision variable will start at *x* = 0, decrease, and then increase again to reach the threshold at zero. However, when *α* < *α*_*s*_, which yields *η* < 0, the patch decision variable will start at zero and will at first decrease, crossing the threshold at an early time *t* < T*, then staying below the threshold before reaching it again at time T*. Therefore, for some range of values *α*_*crit*_ < *α* < *α*_*S*_, there will be two threshold crossings, one at *t* < T* and one at *t* = T*, while outside of this range there is only a single threshold crossing at *t* = T*.

We solve for the critical value of the drift rate, *α*_*crit*_, by considering the derivative of the patch decision variable at *t* = T*. The critical value is when the derivative of the patch decision variable changes signs from positive to negative. Using Eqs 2, 5 and 8, this leads to

$$\begin{array}{c} {\left[\alpha_{crit}-\rho_{0}e^{-T/A}\right]}_{T=T^{*}}=\alpha_{crit}-E-s=0, \end{array}$$ (18)

which yields *α*_*crit*_ = *E* + *s*. For drift values in the range *α*_*crit*_ < *α* < *α*_*S*_, there will be two threshold crossings, and therefore a simulation would need an extra rule to “ignore” the first crossing in order to obtain optimal decisions. We therefore restrict drift values to be outside of this range. In our analysis, we make a further restriction to simplify results by additionally neglecting the range 0 < *α* < *α*_*crit*_, because in this range *α* and *η* have opposite signs. Note that when *α* is near the boundaries of this range, we can expect patch decisions to be very sensitive to the addition of noise on the patch decision variable, uncertainty in patch characteristics, and/or if rewards come in discrete chunks.

### Drift and threshold choices for optimal patch residence times with patch uncertainty

When patches vary in food density and size, we use the average initial patch food density, $\bar{\rho_{0}}$, and the average patch size, $\bar{A}$, to define values of the drift rate, *α*, and the threshold, *η*. Here we derive expressions for *α* and *η* to consider two possible cases: to optimally adjust patch residence times for uncertainty in patch density, or to optimally adjust patch residence times for uncertainty in patch size.

Eq 8 is the MVT-optimal form for patch residence time as a function of patch density and patch size; we rewrite it here using *E* instead of 〈*E*〉:

$$\begin{array}{c} T^{*}=A\ln\frac{\rho_{0}}{E+s}. \end{array}$$ (19)

Consider a small change of patch residence time of the form

$$\begin{array}{c} T=T^{*}+\delta T. \end{array}$$ (20)

To determine the optimal drift rate for uncertainty in patch food density, now consider a small change in patch density about an average value via the expansion $\rho_{0}=\bar{\rho_{0}}+\delta \rho_{0}$. Plugging this into Eq 19, expanding to first order terms, and comparing with Eq 20 yields the optimal first order changes in patch residence time as function of changes in individual patch density:

$$\begin{array}{c} \delta T^{*}=\frac{\bar{A}}{\bar{\rho_{0}}}\delta \rho_{0}. \end{array}$$ (21)

Similarly, considering a change in patch size of the form $A=\bar{A}+\delta A$ yields an optimal first order change in patch residence time with changes in patch size:

$$\begin{array}{c} \delta T^{*}=\ln\frac{\bar{\rho_{0}}}{E+s}\delta A. \end{array}$$ (22)

We derive values for the drift rate and threshold so that either Eq 21 or Eq 22 are satisfied; these represent two different strategies that an animal may use to adapt to uncertainty in an environment. In doing so, we demonstrate that both Eqs 21 and 22 cannot be satisfied; the strategies represented by these cases represent a tradeoff between optimally adapting to uncertainty in patch density versus optimally adapting to uncertainty in patch size.

Start with the integral of the patch decision variable equation (Eq 2) with zero noise, using the average reward rate from Eq 5. Then, integrating up to a time *T* when the threshold is reached yields

$$\begin{array}{c} \eta =\alpha T+\rho_{0}A(e^{-T/A}-1) \end{array}$$ (23)

Applying the condition that the threshold is reached at the MVT-optimal patch residence time in Eq 19 yields a relationship between the threshold and the drift rate:

$$\begin{array}{c} \eta =\bar{A}(\alpha \ln(\frac{\bar{\rho_{0}}}{E+s})-\bar{\rho_{0}}+E+s), \end{array}$$ (24)

where we note that this is the same form as Eq 9, except that here the average patch parameters $\bar{A}$ and $\bar{\rho_{0}}$ are used. We now combine Eqs 23 and 24, plug in expansions for *T* = T* + *δT* and $\rho_{0}=\bar{\rho_{0}}+\delta \rho_{0}$, expand to first order in *δT*, and solve for the first-order changes in patch residence times:

$$\begin{array}{cc} \delta T & =\frac{\delta \rho_{0}\bar{A}(-\bar{\rho_{0}}+E+s)}{\bar{\rho_{0}}\left(-\alpha +E+s\right)+\delta \rho_{0}\left(E+s\right)}\\ & \approx \frac{\bar{A}(-\bar{\rho_{0}}+E+s)}{\bar{\rho_{0}}\left(-\alpha +E+s\right)}\delta \rho_{0}, \end{array}$$ (25)

where the approximation uses a series expansion in *δρ*_0_ to first order terms. Comparing this with Eq 21 leads a value of *α* which satisfies optimal adaptation to uncertainty in patch density, which is simply

$$\begin{array}{c} \alpha =\bar{\rho_{0}}. \end{array}$$ (26)

We use an analogous process to calculate values of the drift rate and threshold for optimal adaptation to uncertainty in patch size. Again we combine Eqs 23 and 24, then plug in expansions for *T* = T* + *δT* and $A=\bar{A}+\delta A$, expand to first order in *δT*, and solve for the first-order changes in patch residence times:

$$\begin{array}{cc} \delta T & =\frac{\bar{\rho_{0}}(\bar{A}+\delta A)-(\frac{\bar{\rho_{0}}}{E+s}){}^{\frac{\bar{A}}{\bar{A}+\delta A}}(\left(E+s\right)\bar{A}+\bar{\rho_{0}}\delta A)}{\bar{\rho_{0}}-\alpha (\frac{\bar{\rho_{0}}}{E+s}){}^{\frac{\bar{A}}{\bar{A}+\delta A}}}\\ & \approx \frac{\left(E+s\right)(\ln(\frac{\bar{\rho_{0}}}{E+s})+1)-\bar{\rho_{0}}}{-\alpha +E+s}\delta A, \end{array}$$ (27)

where the approximation uses a series expansion in *δA* to first order terms. Comparing this with Eq 22 and solving for *α* yields the drift rate that satisfies optimal adaptation to uncertainty in patch size:

$$\begin{array}{c} \alpha =\frac{\bar{\rho_{0}}-e-s}{\ln(\frac{\bar{\rho_{0}}}{E+s})}. \end{array}$$ (28)

Using this in Eq 24 yields the threshold value of *η* = 0. Thus, for optimal adaptation to patch size, the decision variable will start at zero, decrease to negative values as the animal finds food, and then increase back to zero for a decision to leave the patch.

## Supporting information

S1 Fig Full simulation results with added patch decision noise. Shown are the average and standard deviation of the energy intake (left grid) and patch residence times (right grid), for the density adaptive strategy (top) and the robust counting strategy (bottom), when the noise on the patch decision variable (*σ*) is increased. The robust counting strategy is implemented by setting *α* = −0.2*ρ*_0_ for each case. Each grid of 9 plots contains simulation results with different values of the travel time and the MVT-optimal available energy in the environment: columns correspond to values of *T*_*tr*_ = (1, 5, 10)*τ*, and rows correspond to values of E* = (0.5, 2, 5)*s*. For each plot, the filled blue curve uses a patch size of *A* = 1.5*τ*, the filled red curve uses a patch size of *A* = 5*τ*, and solid line is the MVT-optimal energy or patch time.
(EPS)

S2 Fig Full simulation results with discrete food rewards. The organization of the grid of plots and other parameters are the same as S1 Fig, but shown here are simulation results when the food chunks size (*c*) is increased.
(EPS)

S3 FigUncertain patch size and adaptive strategies.Shown are simulations in an environment where the patch size is uncertain. The size of individual patches, *A*, is drawn from a Gaussian distribution with mean $\bar{A}=5$ and standard deviation $\Delta A=0.3\bar{A}$. The average energy and patch residence times, and the distribution of individual patch residence times, are shown for three strategies: the density adaptive and robust counting strategies are implemented in the same manner as in Fig 4, and also an approximate size-adaptive strategy with *α* = 1.05*α*_*S*_, where *α*_*S*_ is the drift value for the size-adaptive strategy. Other parameters are set corresponding to Fig 4: *T*_*tr*_ = 5, E* = 2 (or equivalently, *ρ*_0_ = 9.439), *c* = 0, and *σ* = 0.3*ρ*_0_. The bottom three plots show patch residence times for each strategy along with the MVT-optimal relationship from Eq 8, and the approximate adjustment to PRTs calculated in Eq 27 according to the value of *α* for each strategy.(EPS)

S4 FigFull simulation results with different strategies and forms of the marginal utility function.Analogous results to Fig 5C and 5D are shown here for both the density-adaptive strategy (left grid) and the robust counting strategy (right grid), each in the two environments from Fig 4: uncertain patch food density (top row), and scattered patches with discrete reward (bottom row). Simulation parameters correspond to the analogous cases in Fig 4, except for the available energy in the environment, which is varied here by changing the value of $\bar{\rho_{0}}$ in the simulations. For each case of the utility function, the average energy intake and patch residence time are shown for two different values of *β*. Solid lines are an approximate solution to the governing equations and points are the mean and standard deviation of simulation results. (A) Results using the exponential marginal utility function (see Fig 5A). (B) Results using the linear threshold marginal utility function (see Fig 5B).(EPS)

S1 Appendix Fokker-Planck formulation and numerical solution for probability density. (PDF)

S2 Appendix Optimal energy when patches vary in quality. (PDF)

## References

[1] Stone M. Models for choice-reaction time. Psychometrika. 1960;25(3):251–260. doi: 10.1007/BF02289729

[2] Laming DRJ. Information theory of choice-reaction times. Academic Press; 1968.

[3] Link SW. The relative judgment theory of two choice response time. Journal of Mathematical Psychology. 1975;12(1):114–135. doi: 10.1016/0022-2496(75)90053-X

[4] Ratcliff R, McKoon G. The diffusion decision model: theory and data for two-choice decision tasks. Neural computation. 2008;20(4):873–922. doi: 10.1162/neco.2008.12-06-420 18085991 PMC2474742

[5] Falmagne J. Stochastic models for choice reaction time with applications to experimental results. Journal of Mathematical Psychology. 1965;2(1):77–124. doi: 10.1016/0022-2496(65)90018-0

[6] Bogacz R, Brown E, Moehlis J, Holmes P, Cohen JD. The physics of optimal decision making: a formal analysis of models of performance in two-alternative forced-choice tasks. Psychological review. 2006;113(4):700. doi: 10.1037/0033-295X.113.4.700 17014301

[7] Piet AT, El Hady A, Brody CD. Rats adopt the optimal timescale for evidence integration in a dynamic environment. Nature communications. 2018;9(1):4265. doi: 10.1038/s41467-018-06561-y 30323280 PMC6189050

[8] Krajbich I, Lu D, Camerer C, Rangel A. The attentional drift-diffusion model extends to simple purchasing decisions. Frontiers in psychology. 2012;3:193. doi: 10.3389/fpsyg.2012.00193 22707945 PMC3374478

[9] Brunton BW, Botvinick MM, Brody CD. Rats and Humans Can Optimally Accumulate Evidence for Decision-Making. Science. 2013;340(6128):95–98. doi: 10.1126/science.1233912 23559254

[10] Hanks TD, Kopec CD, Brunton BW, Duan CA, Erlich JC, Brody CD. Distinct relationships of parietal and prefrontal cortices to evidence accumulation. Nature. 2015;advance online publication. doi: 10.1038/nature14066 25600270 PMC4835184

[11] Gluth S, Rieskamp J, Büchel C. Deciding when to decide: time-variant sequential sampling models explain the emergence of value-based decisions in the human brain. Journal of Neuroscience. 2012;32(31):10686–10698. doi: 10.1523/JNEUROSCI.0727-12.2012 22855817 PMC6621398

[12] Ratcliff R. A theory of memory retrieval. Psychological review. 1978;85(2):59. doi: 10.1037/0033-295X.85.2.59

[13] Purcell BA, Heitz RP, Cohen JY, Schall JD, Logan GD, Palmeri TJ. Neurally constrained modeling of perceptual decision making. Psychological review. 2010;117(4):1113. doi: 10.1037/a0020311 20822291 PMC2979343

[14] Milosavljevic M, Malmaud J, Huth A, Koch C, Rangel A. The Drift Diffusion Model can account for the accuracy and reaction time of value-based choices under high and low time pressure. Judgment and Decision Making. 2010;5(6):437–449.

[15] Deco G, Rolls ET, Albantakis L, Romo R. Brain mechanisms for perceptual and reward-related decision-making. Prog in Neurobiol. 2013;103:194–213. doi: 10.1016/j.pneurobio.2012.01.010 22326926

[16] Doya K, (Eds) S MN. Decision Making. Curr Opin Neurobiol. 2012;22 (6). 10.1016/j.conb.2012.10.003 23177659

[17] Kim JN, Shadlen MN. Neural correlates of a decision in the dorsolateral prefrontal cortex of the macaque. Nat Neurosi. 1999;2:176–185. doi: 10.1038/5739 10195203

[18] Horwitz GD, Newsome WT. Representation of an abstract perceptual decision in macaque superior colliculus. J Neurophysiol. 2004;91:2281–2296. doi: 10.1152/jn.00872.2003 14711971

[19] Ding L, Gold JI. Caudate encodes multiple computations for perceptual decisions. J Neurosci. 2010;30:15747–15759. doi: 10.1523/JNEUROSCI.2894-10.2010 21106814 PMC3005761

[20] Ding L, Gold JI. Neural correlates of perceptual decision making before, during, and after decision commitment in monkey frontal eye field. Cereb Cortex. 2012;22:1052–1067. doi: 10.1093/cercor/bhr178 21765183 PMC3328342

[21] Ding L, Gold JI. Separate, causal roles of the caudate in saccadic choice and execution in a perceptual decision task. Neuron. 2012;75:865–874. doi: 10.1016/j.neuron.2012.07.021 22958826 PMC3446771

[22] Churchland AK, Kiani R, Chaudhuri R, Wang XJ, Pouget A, Shadlen MN. Variance as a signature of neural computations during decision making. Neuron. 2011;69(4):818–831. doi: 10.1016/j.neuron.2010.12.037 21338889 PMC3066020

[23] Huk AC, Shadlen MN. Neural activity in macaque parietal cortex reflects temporal integration of visual motion signals during perceptual decision making. Journal of Neuroscience. 2005;25(45):10420–10436. doi: 10.1523/JNEUROSCI.4684-04.2005 16280581 PMC6725829

[24] Evans DA, Stempel AV, Vale R, Ruehle S, Lefler Y, Branco T. A synaptic threshold mechanism for computing escape decisions. Nature. 2018;558(7711):590. doi: 10.1038/s41586-018-0244-6 29925954 PMC6235113

[25] Krakauer JW, Ghazanfar AA, Gomez-Marin A, MacIver MA, Poeppel D. Neuroscience Needs Behavior: Correcting a Reductionist Bias. Neuron. 2017;93(3):480–490. doi: 10.1016/j.neuron.2016.12.041 28182904

[26] Mobbs D, Trimmer PC, Blumstein DT, Dayan P. Foraging for foundations in decision neuroscience: insights from ethology. neuroscience. 2018;13(18):19. 10.1038/s41583-018-0010-7 PMC6786488 29752468

[27] Stephens DW, Krebs JR. Foraging theory. Princeton University Press; 1986.

[28] Charnov EL. Optimal foraging, the marginal value theorem. Theoretical population biology. 1976;9(2):129–136. doi: 10.1016/0040-5809(76)90040-X 1273796

[29] Nonacs P. State dependent behavior and the marginal value theorem. Behavioral Ecology. 2001;12(1):71–83. doi: 10.1093/oxfordjournals.beheco.a000381

[30] Goldstein DG, Gigerenzer G. Models of ecological rationality: the recognition heuristic. Psychological review. 2002;109(1):75. doi: 10.1037/0033-295X.109.1.75 11863042

[31] Todd PM, Gigerenzer G. Environments that make us smart: Ecological rationality. Current directions in psychological science. 2007;16(3):167–171. doi: 10.1111/j.1467-8721.2007.00497.x

[32] Hayden BY, Pearson JM, Platt ML. Neuronal basis of sequential foraging decisions in a patchy environment. Nature neuroscience. 2011;14(7):933. doi: 10.1038/nn.2856 21642973 PMC3553855

[33] Shenhav A, Straccia MA, Cohen JD, Botvinick MM. Anterior cingulate engagement in a foraging context reflects choice difficulty, not foraging value. Nature neuroscience. 2014;17(9):1249. doi: 10.1038/nn.3771 25064851 PMC4156480

[34] Calhoun AJ, Chalasani SH, Sharpee TO. Maximally informative foraging by Caenorhabditis elegans. Elife. 2014;3. doi: 10.7554/eLife.04220 25490069 PMC4358340

[35] Calhoun AJ, Hayden BY. The foraging brain. Current Opinion in Behavioral Sciences. 2015;5:24–31. doi: 10.1016/j.cobeha.2015.07.003

[36] Hayden BY, Walton ME. Neuroscience of foraging. Frontiers in neuroscience. 2014;8:81. doi: 10.3389/fnins.2014.00081 24795556 PMC4001030

[37] Li F, Li M, Cao W, Xu Y, Luo Y, Zhong X, et al. Anterior cingulate cortical lesion attenuates food foraging in rats. Brain research bulletin. 2012;88(6):602–608. doi: 10.1016/j.brainresbull.2012.05.015 22683801

[38] Barack DL, Chang SW, Platt ML. Posterior cingulate neurons dynamically signal decisions to disengage during foraging. Neuron. 2017;96(2):339–347. doi: 10.1016/j.neuron.2017.09.048 29024659 PMC5788808

[39] Waage JK. Foraging for patchily-distributed hosts by the parasitoid, Nemeritis canescens. The Journal of Animal Ecology. 1979; p. 353–371. doi: 10.2307/4166

[40] McNamara J. Optimal patch use in a stochastic environment. Theoretical Population Biology. 1982;21(2):269–288. doi: 10.1016/0040-5809(82)90018-1

[41] Driessen G, Bernstein C. Patch departure mechanisms and optimal host exploitation in an insect parasitoid. Journal of Animal Ecology. 1999;68(3):445–459. doi: 10.1046/j.1365-2656.1999.00296.x

[42] Haccou P, De Vlas SJ, Van Alphen JJ, Visser ME. Information processing by foragers: effects of intra-patch experience on the leaving tendency of Leptopilina heterotoma. The Journal of Animal Ecology. 1991; p. 93–106. doi: 10.2307/5447

[43] Taneyhill DE. Patch departure behavior of bumble bees: rules and mechanisms. Psyche: A Journal of Entomology. 2010;2010.

[44] Smith PL, Ratcliff R. Psychology and neurobiology of simple decisions. Trends in Neurosciences. 2004;27(3):161–168. doi: 10.1016/j.tins.2004.01.006 15036882

[45] Ratcliff R, Smith PL, Brown SD, McKoon G. Diffusion decision model: current issues and history. Trends in cognitive sciences. 2016;20(4):260–281. doi: 10.1016/j.tics.2016.01.007 26952739 PMC4928591

[46] Ward JF, Austin RM, Macdonald DW. A simulation model of foraging behaviour and the effect of predation risk. Journal of Animal Ecology. 2000;69(1):16–30. doi: 10.1046/j.1365-2656.2000.00371.x

[47] Zhang F, Hui C. Recent experience-driven behaviour optimizes foraging. Animal behaviour. 2014;88:13–19. doi: 10.1016/j.anbehav.2013.11.002

[48] Wajnberg E, Fauvergue X, Pons O. Patch leaving decision rules and the Marginal Value Theorem: an experimental analysis and a simulation model. Behavioral Ecology. 2000;11(6):577–586. doi: 10.1093/beheco/11.6.577

[49] Iwasa Y, Higashi M, Yamamura N. Prey distribution as a factor determining the choice of optimal foraging strategy. The American Naturalist. 1981;117(5):710–723. doi: 10.1086/283754

[50] Real L, Caraco T. Risk and foraging in stochastic environments. Annual Review of Ecology and Systematics. 1986;17(1):371–390. doi: 10.1146/annurev.es.17.110186.002103

[51] Simon HA. Theories of decision-making in economics and behavioral science. The American economic review. 1959;49(3):253–283.

[52] Simon HA, et al. An empirically-based microeconomics. Cambridge Books. 2009.

[53] Ward D. The role of satisficing in foraging theory. Oikos. 1992; p. 312–317. doi: 10.2307/3545394

[54] Nonacs P, Dill LM. Is satisficing an alternative to optimal foraging theory? Oikos. 1993; p. 371–375. doi: 10.2307/3545484

[55] Rita H, Ranta E. Stochastic patch exploitation model. Proceedings of the Royal Society of London B: Biological Sciences. 1998;265(1393):309–315. doi: 10.1098/rspb.1998.0297

[56] Eliassen S, Jørgensen C, Mangel M, Giske J. Quantifying the Adaptive Value of Learning in Foraging Behavior. The American Naturalist. 2009;174(4):478–489. doi: 10.1086/605370 19694535

[57] Ratcliff R, Smith PL. A comparison of sequential sampling models for two-choice reaction time. Psychological review. 2004;111(2):333. doi: 10.1037/0033-295X.111.2.333 15065913 PMC1440925

[58] Kagel JH, Green L, Caraco T. When foragers discount the future: Constraint or adaptation? Animal Behaviour. 1986;34:271–283. doi: 10.1016/0003-3472(86)90032-1

[59] Stephens DW. Decision ecology: foraging and the ecology of animal decision making. Cognitive, Affective, & Behavioral Neuroscience. 2008;8(4):475–484. doi: 10.3758/CABN.8.4.475 19033242

[60] Hayden BY. Time discounting and time preference in animals: a critical review. Psychonomic bulletin & review. 2016;23(1):39–53. doi: 10.3758/s13423-015-0879-3 26063653

[61] Blanchard TC, Pearson JM, Hayden BY. Postreward delays and systematic biases in measures of animal temporal discounting. Proceedings of the National Academy of Sciences. 2013; p. 201310446. doi: 10.1073/pnas.1310446110 PMC3780845 24003113

[62] Constantino SM, Daw ND. Learning the opportunity cost of time in a patch-foraging task. Cognitive, Affective, & Behavioral Neuroscience. 2015;15(4):837–853. doi: 10.3758/s13415-015-0350-y PMC4624618 25917000

[63] Carter EC, Redish AD. Rats value time differently on equivalent foraging and delay-discounting tasks. Journal of Experimental Psychology: General. 2016;145(9):1093. doi: 10.1037/xge0000196 27359127 PMC5050558

[64] Wikenheiser AM, Stephens DW, Redish AD. Subjective costs drive overly patient foraging strategies in rats on an intertemporal foraging task. Proceedings of the National Academy of Sciences. 2013; p. 201220738. doi: 10.1073/pnas.1220738110 PMC3657802 23630289

[65] Kacelnik A, Vasconcelos M, Monteiro T, Aw J. Darwin’s “tug-of-war” vs. starlings’ “horse-racing”: how adaptations for sequential encounters drive simultaneous choice. Behavioral Ecology and Sociobiology. 2011;65(3):547–558. doi: 10.1007/s00265-010-1101-2

[66] Gomez P, Ratcliff R, Perea M. A Model of the Go/No-Go Task. Journal of experimental psychology General. 2007;136(3):389–413. doi: 10.1037/0096-3445.136.3.389 17696690 PMC2701630

[67] Stephens DW, Brown JS, Ydenberg RC. Foraging: behavior and ecology. University of Chicago Press; 2008.

[68] Greene JS, Brown M, Dobosiewicz M, Ishida IG, Macosko EZ, Zhang X, et al. Balancing selection shapes density-dependent foraging behaviour. Nature. 2016;539(7628):254–258. doi: 10.1038/nature19848 27799655 PMC5161598

[69] Scholz M, Dinner AR, Levine E, Biron D. Stochastic feeding dynamics arise from the need for information and energy. Proceedings of the National Academy of Sciences. 2017;114(35):9261–9266. doi: 10.1073/pnas.1703958114 PMC5584422 28802256

[70] Strandburg-Peshkin A, Farine DR, Couzin ID, Crofoot MC. Shared decision-making drives collective movement in wild baboons. Science. 2015;348(6241):1358–1361. doi: 10.1126/science.aaa5099 26089514 PMC4801504

[71] Srivastava V, Leonard NE. Collective Decision-Making in Ideal Networks: The Speed-Accuracy Tradeoff. IEEE Transactions on Control of Network Systems. 2014;1(1):121–132. doi: 10.1109/TCNS.2014.2310271

[72] Kolling N, Akam T. (Reinforcement?) Learning to forage optimally. Current Opinion in Neurobiology. 2017;46:162–169. doi: 10.1016/j.conb.2017.08.008 28918312

[73] McNamara J, Houston A. The application of statistical decision theory to animal behaviour. Journal of Theoretical Biology. 1980;85(4):673–690. doi: 10.1016/0022-5193(80)90265-9 7442286

[74] Krakauer DC, Rodriguez-Gironés MA. Searching and Learning in a Random Environment. Journal of Theoretical Biology. 1995;177(4):417–429. doi: 10.1006/jtbi.1995.0258

[75] Biernaskie JM, Walker SC, Gegear RJ. Bumblebees learn to forage like Bayesians. The American Naturalist. 2009;174(3):413–423. doi: 10.1086/603629 19630548

[76] Marschall EA, Chesson PL, Stein RA. Foraging in a patchy environment: prey-encounter rate and residence time distributions. Animal Behaviour. 1989;37:444–454. doi: 10.1016/0003-3472(89)90091-2

[77] Bartumeus F, Campos D, Ryu WS, Lloret-Cabot R, Méndez V, Catalan J. Foraging success under uncertainty: search tradeoffs and optimal space use. Ecology letters. 2016;19(11):1299–1313. doi: 10.1111/ele.12660 27634051

[78] McNamara JM, Fawcett TW, Houston AI. An adaptive response to uncertainty generates positive and negative contrast effects. Science. 2013;340(6136):1084–1086. doi: 10.1126/science.1230599 23723234

[79] Kamil AC, Misthal RL, Stephens DW. Failure of simple optimal foraging models to predict residence time when patch quality is uncertain. Behavioral Ecology. 1993;4(4):350–363. doi: 10.1093/beheco/4.4.350

[80] Higginson AD, Fawcett TW, Trimmer PC, McNamara JM, Houston AI. Generalized Optimal Risk Allocation: Foraging and Antipredator Behavior in a Fluctuating Environment. The American Naturalist. 2012;180(5):589–603. doi: 10.1086/667885 23070320

[81] Blanchard T, Wilke A, Hayden B. Hot-Hand Bias in Rhesus Monkeys. Journal of Experimental Psychology: Animal Learning and Cognition. 2014;40:280. 25545977 10.1037/xan0000033

[82] Kacelnik A, Todd IA. Psychological mechanisms and the marginal value theorem: effect of variability in travel time on patch exploitation. Animal Behaviour. 1992;43(2):313–322. doi: 10.1016/S0003-3472(05)80226-X

[83] Cuthill IC, Kacelnik A, Krebs JR, Haccou P, Iwasa Y. Starlings exploiting patches: the effect of recent experience on foraging decisions. Animal Behaviour. 1990;40(4):625–640. doi: 10.1016/S0003-3472(05)80692-X

[84] Thiel A, Hoffmeister TS. Knowing your habitat: linking patch-encounter rate and patch exploitation in parasitoids. Behavioral Ecology. 2004;15(3):419–425. doi: 10.1093/beheco/arh030

[85] Wildhaber ML, Green RF, Crowder LB. Bluegills continuously update patch giving-up times based on foraging experience. Animal Behaviour. 1994;47(3):501–513. doi: 10.1006/anbe.1994.1075

[86] Outreman Y, Le Ralec A, Wajnberg E, Pierre JS. Effects of within- and among-patch experiences on the patch-leaving decision rules in an insect parasitoid. Behavioral Ecology and Sociobiology. 2005;58(2):208–217. doi: 10.1007/s00265-004-0895-1

[87] Thiel A, Hoffmeister T. Selective information use in parasitoid wasps. Animal Biology. 2006;56(2):233–245. doi: 10.1163/157075606777304212

[88] Kerr JN, Nimmerjahn A. Functional imaging in freely moving animals. Current opinion in neurobiology. 2012;22(1):45–53. doi: 10.1016/j.conb.2011.12.002 22237048

[89] Yin M, Borton DA, Komar J, Agha N, Lu Y, Li H, et al. Wireless neurosensor for full-spectrum electrophysiology recordings during free behavior. Neuron. 2014;84(6):1170–1182. doi: 10.1016/j.neuron.2014.11.010 25482026

[90] Wiltschko AB, Johnson MJ, Iurilli G, Peterson RE, Katon JM, Pashkovski SL, et al. Mapping sub-second structure in mouse behavior. Neuron. 2015;88(6):1121–1135. doi: 10.1016/j.neuron.2015.11.031 26687221 PMC4708087

[91] El Hady A. Closed loop neuroscience. Academic Press; 2016.

