# Supplementary material for: Foraging as an evidence accumulation process
Source: PLoS Comput Biol. 2019 Jul 24;15(7):e1007060. doi: 10.1371/journal.pcbi.1007060 (PMC6682163; doi:10.1371/journal.pcbi.1007060)
Supplement: S1 Appendix — (PDF) [file pcbi.1007060.s005.pdf]

Supplementary material

S1 Fokker-Planck formulation and numerical solution for probability density

Consider the stochastic differential equation for the patch decision variable, rewritten here for clarity:

$$\tau dx = (\alpha - r(t))\, dt + \sigma dW(t). \tag{S1}$$

We will formulate this as a Fokker-Planck equation and solve for the probability density via the finite element method. To do this, we first define a normalized patch decision variable with

$$y = \tau \frac{x(t)}{\eta(t)}, \tag{S2}$$

and take the differential:

$$\begin{aligned} dy &= \tau \frac{dx(t)}{\eta(t)} - \tau \frac{x(t)}{\eta(t)^2} d\eta(t) \\ &\approx \tau \frac{dx(t)}{\eta(t)}, \end{aligned} \tag{S3}$$

where the approximation is used, because the threshold $\eta(t)$ changes slowly compared to the patch decision variable. Now we can write a new equation with this change of variables:

$$dy = (\alpha_y(t) - r_y(t))\, dt + \sigma_y(t) dW(t), \tag{S4}$$

where $\alpha_y(t) \equiv \alpha/\eta(t)$, $r_y(t) \equiv r(t)/\eta(t)$, and $\sigma_y(t) \equiv \sigma/\eta(t)$, and the decision threshold occurs at $y = 1$. Note that since we consider strategies where α and η are either zero or have the same sign, $\alpha_y(t)$ will always be either zero or positive, setting a drift towards the threshold. For food rewards, if $\eta > 0$ then $r_y > 0$, and from Eq. S4 food reward will decrease y, i.e. lowering it away from the threshold of $y = 1$. If $\eta < 0$, then $r_y < 0$, and food will increase y towards the threshold. Thus, the normalized formulation with the threshold at $y = 1$ can represent the different decisions strategies without any other further modifications.

The Fokker-Planck equation corresponding to Eq. S4 is

$$\frac{\partial G}{\partial t} = -\left(\alpha_y(t) - r_y(t)\right) \frac{\partial G}{\partial y} + \frac{\sigma_y(t)^2}{2} \frac{\partial^2 G}{\partial y^2}, \tag{S5}$$

where $G(y, t)$ is the time-dependent probability density for the normalized decision variable y. We keep the terms $\alpha_y(t)$ and $r_y(t)$ separate, because the former is a continuous function while the latter is defined by discrete inputs via a Poisson process when food rewards are received in chunks.

To solve this using the finite element method, first let $G = N_i(y) g_i(t)$, where $N_i(y)$ are the shape functions and $g_i(t)$ are the nodal variables. Summation notation applies over the indices i and j. After writing the weak form of the equation and setting the integral of the residual to zero, we obtain the finite element matrix equation:

$$M_{ij} \frac{dg_j}{dt} = -\left(\alpha_y(t) - r_y(t)\right) B_{ij} g_j + \frac{\sigma_y(t)^2}{2} A_{ij} g_j, \tag{S6}$$

where M_{ij} is the mass matrix, A_{ij} is a second-derivative matrix operator, and B_{ij} is a first-derivative matrix operator. We consider the solution over a domain of $[-L, 1]$, and choose the lower value of the domain as sufficiently low to encompass the full range of the probability distribution of y. The upper boundary of $y = 1$ is absorbing, and therefore has the condition $G(1, t) = 0$. We define the lower boundary as reflecting: $\partial G(-L, t)/\partial t = 0$.

The mass matrix is defined by integrating the shape functions:

$$M_{ij} = \int_{-L}^{1} N_i N_j dy. \tag{S7}$$

To define A_{ij}, which is the second derivative matrix operator, we will use integration by parts so that only a first derivative remains (and thus we will only need to use linear shape functions). Writing out the integral, and then integrating by parts, we have

$$\begin{aligned} A_{ij} &= \int_{-L}^{1} N_i \frac{\partial^2 N_j}{\partial y^2} dy \\ &= N_i \frac{\partial N_j}{\partial y}\bigg|_{-L}^{1} - \int_{-L}^{1} \frac{\partial N_i}{\partial y} \frac{\partial N_j}{\partial y} dy \\ &= N_i \frac{\partial N_j}{\partial y}\bigg|^{1} - \int_{-L}^{1} \frac{\partial N_i}{\partial y} \frac{\partial N_j}{\partial y} dy, \end{aligned} \tag{S8}$$

where the last equality uses the zero-flux reflecting boundary condition at $y = -L$. For all elements the 2nd term in Eq. S8 yields $1/dy((-1, 1), (1, -1))$, where dy is the size of each element. The absorbing boundary at $y = 1$ leads to a nonzero flux, and therefore must be included in the global matrix calculation. To do this, consider the last element in the mesh. Evaluating the boundary term yields an element matrix of $1/dy((0, 0), (1, -1))$, which must also be included in the calculation of A_{ij} to enforce the boundary condition.

The first derivative matrix operator, B_{ij}, is also defined by integrating by parts:

$$\begin{aligned} B_{ij} &= \int_{-L}^{1} N_i \frac{\partial N_j}{\partial y} dy \\ &= N_i N_j|_{-L}^{1} - \int_{-L}^{1} \frac{\partial N_i}{\partial y} N_j dy \\ &= N_i N_j|_{-L} - \int_{-L}^{1} \frac{\partial N_i}{\partial y} N_j dy \end{aligned} \tag{S9}$$

where the last equality applies the absorbing boundary condition of $G(1, t) = 0$. The reflecting boundary condition at $y = -L$ adds an additional contribution of $((1, 0), (0, 0))$ to the first element of the mesh, which must also be included in the calculation of B_{ij}.

To solve these equations numerically, the discrete food rewards are treated separately from the drift and diffusion of the probability density. Therefore, in the code, we solve the equation

$$M_{ij} \frac{dg_j}{dt} = -\alpha_y(t) B_{ij} g_j + \frac{\sigma_y(t)^2}{2} A_{ij} g_j, \tag{S10}$$

and add an extra statement to shift the probability distribution when discrete food rewards $r_y(t)$ are received.

We use the simulation to determine the flux through the upper boundary and the time-dependent probability $P(t)$ that a decision to leave the patch has been made. Flux through the upper boundary can occur from either drift, diffusion, or the receipt of food reward. We calculate $P(t)$ by integrating over the probability density:

$$P(t) = 1 - \int_{-L}^{1} G(y,t)dy. \tag{S11}$$

For the simulations shown in Fig 1C, we coupled patch decisions with the estimate of the energy in the environment by using the expectation value of the decision time:

$$\bar{T} = \int_{0}^{t_{max}} T' P(T') dT'. \tag{S12}$$

where t_{max} is a sufficiently large time value. Alternatively, individual patch decisions could be couple with the energy estimate by sampling from the solution for the probability distribution of patch residence times.
