# Supplementary material for: Foraging as an evidence accumulation process
Source: PLoS Comput Biol. 2019 Jul 24;15(7):e1007060. doi: 10.1371/journal.pcbi.1007060 (PMC6682163; doi:10.1371/journal.pcbi.1007060)
Supplement: S2 Appendix — (PDF) [file pcbi.1007060.s006.pdf]

Supplementary material

S2 Optimal energy when patches vary in quality

To investigate the model dependence on parameters and environmental characteristics, we perform simulations by choosing a value of ρ_0 such that the MVT-optimal energy return of the environment has a certain value. However, when patches vary in quality, we must consider the distribution to calculate the MVT-optimal energy. In the simulation we consider patches where Gaussian noise is added to the initial patch density (ρ_0) and/or the patch size (A). These distributions are defined by mean parameters $\bar{\rho_0}$ and $\bar{A}$ and standard deviation parameters $\Delta\rho_0$ and ΔA. In this section we calculate a correction to the MVT-optimal energy that depends on the standard deviation of initial patch food density $\Delta\rho_0$.

First, we write the average energy in the environment, following Eq. 7:

$$\langle E\rangle = \frac{\left\langle \int_0^{T^*} r(t)dt \right\rangle - s*(T_{tr} + \langle T^*\rangle)}{T_{tr} + \langle T^*\rangle}, \tag{S1}$$

where the average over the environment, denoted $\langle\cdot\rangle$, must be evaluated over the distribution of patches. Using Gaussian probability distributions for these, we have

$$P(\rho_0) = C_{\rho_0} e^{-\frac{(\rho_0-\bar{\rho_0})^2}{2\Delta\rho_0^2}} \tag{S2}$$

$$P(A) = C_A e^{-\frac{(A-\bar{A})^2}{2\Delta A^2}}, \tag{S3}$$

where C_{ρ_0} and C_A are normalization factors. The average of some quantity z over these probability distributions is

$$\langle z\rangle = \int_0^\infty \int_0^\infty z P(\rho_0)P(A)d\rho_0 dA, \tag{S4}$$

where the lower end of the integral should be restricted to zero, because patches cannot have negative density or size. We first use this to evaluate the average return from patches by using the MVT-optimal time from Eq. 8:

$$\begin{aligned}\left\langle \int_0^{T^*} r(t)dt \right\rangle &= \langle \rho_0 A - A\left(\langle E\rangle + s\right)\rangle \\ &\approx \bar{\rho_0}\bar{A} - \bar{A}\left(\langle E\rangle + s\right),\end{aligned} \tag{S5}$$

where the approximation uses an evaluation of the Gaussian probability distribution over a full range, instead of restricting to positive values as expressed in Eq. S4. This approximation holds well for $\Delta\rho_0/\bar{\rho_0} \ll 1$ and $\Delta A/\bar{A} \ll 1$. To evaluate the average MVT-optimal patch residence time, we use the same approximation for the distribution of A, but evaluate the distribution of ρ_0 over the restricted range due to the nonlinear form:

$$\begin{aligned}\langle T^*\rangle &= \left\langle A \ln \frac{\rho_0}{\langle E\rangle + s} \right\rangle \\ &\approx \bar{A}\int_0^\infty \ln \frac{\rho_0}{\langle E\rangle + s} P(\rho_0)d\rho_0.\end{aligned} \tag{S6}$$

The solution to this integral can be expressed in closed form using special functions; we performed this calculation using Mathematica. We use this to calculate a correction to the MVT-optimal energy, which was used to plot results in Fig 4, 5, and Fig **??**. For example, using $\bar{\rho_0} = 9.439$ and $\Delta\rho_0 = 0$ leads to E^*=2, while using $\bar{\rho_0} = 9.439$ and $\Delta\rho_0 = 0.3\bar{\rho_0}$ (which was used Fig 4), leads to $E^* = 2.077$. To apply this to the cases shown in Figs 5 and **??**, we found that the solution for the correction to the MVT-optimal energy, using Eqs. S1 and S6 can be approximated as a linear function function of the "uncorrected" energy, E_0, using $E^* \approx 0.02563 + 1.02563E_0$.
